# Supplementary material for: Tridimensional infiltration of DNA viruses into the host genome shows preferential contact with active chromatin
Source: Nat Commun. 2018 Oct 15;9:4268. doi: 10.1038/s41467-018-06739-4 (PMC6189100; doi:10.1038/s41467-018-06739-4)
Supplement: Supplementary file 1 — Supplementary Information [file 41467_2018_6739_MOESM1_ESM.pdf]

Tridimensional infiltration of DNA viruses into the host genome shows preferential contact with active chromatin

Pierrick Moreau et al.

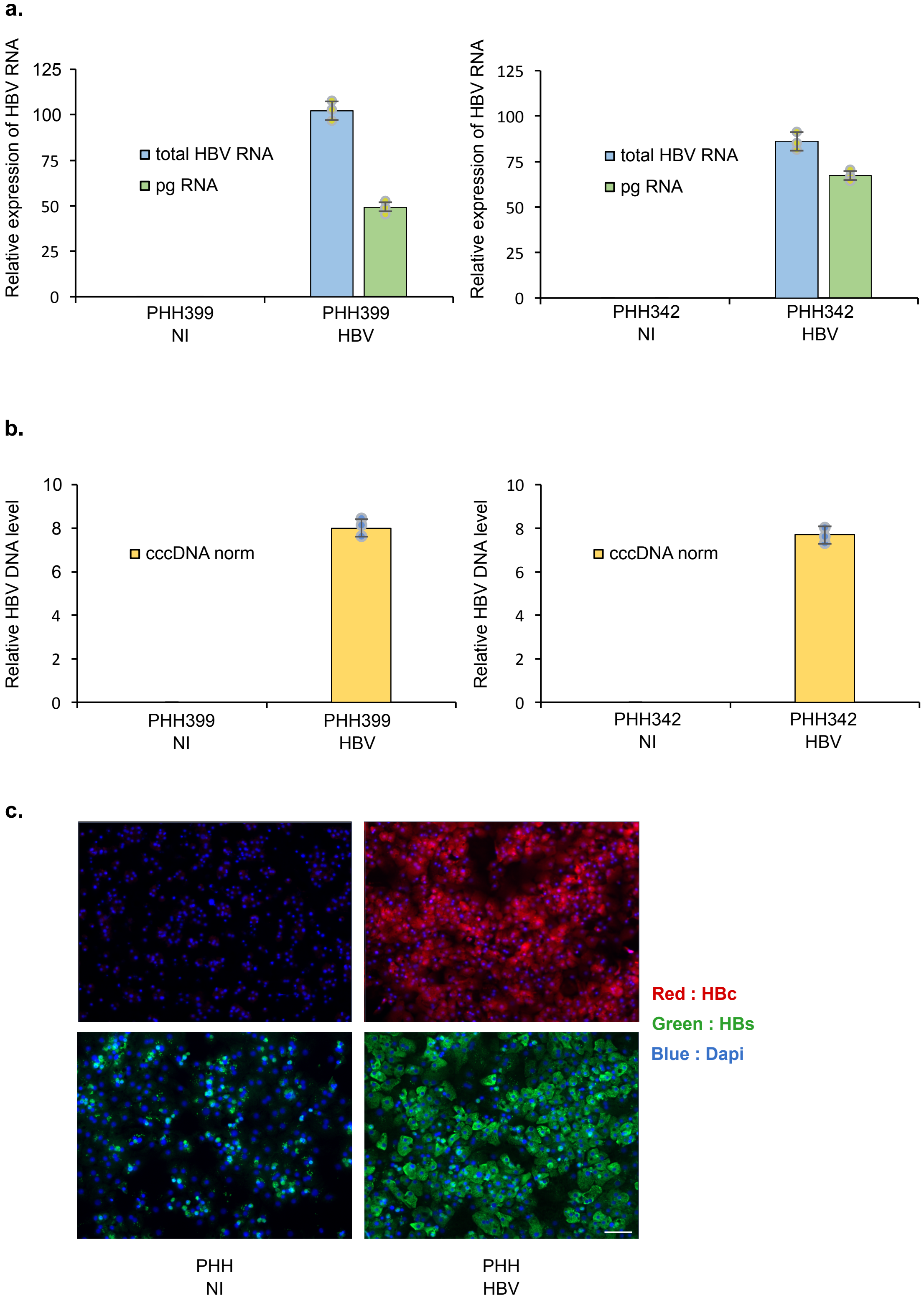

Supplementary Figure 1. PHH (donor 399 or 342) were infected at a MOI of 500 genomes vp/cells with HBV and treated with lamivudine the last 3 days of the experiment. 7 days after infection cells were harvested and analyzed for HBV replication. (a) HBV transcription was assessed by the quantification of total HBV RNA and pgRNA by RT-qPCR. Error bars (n = 3) represent SEM. (b) HBV cccDNA establishment was quantified by qPCR. Error bars (n = 3) represent SEM. (c) HBV capsid protein (Hbc, red) and HBV envelop protein (HBs, green) were visualized using immunofluorescence. Scale bar represents 50 µm.

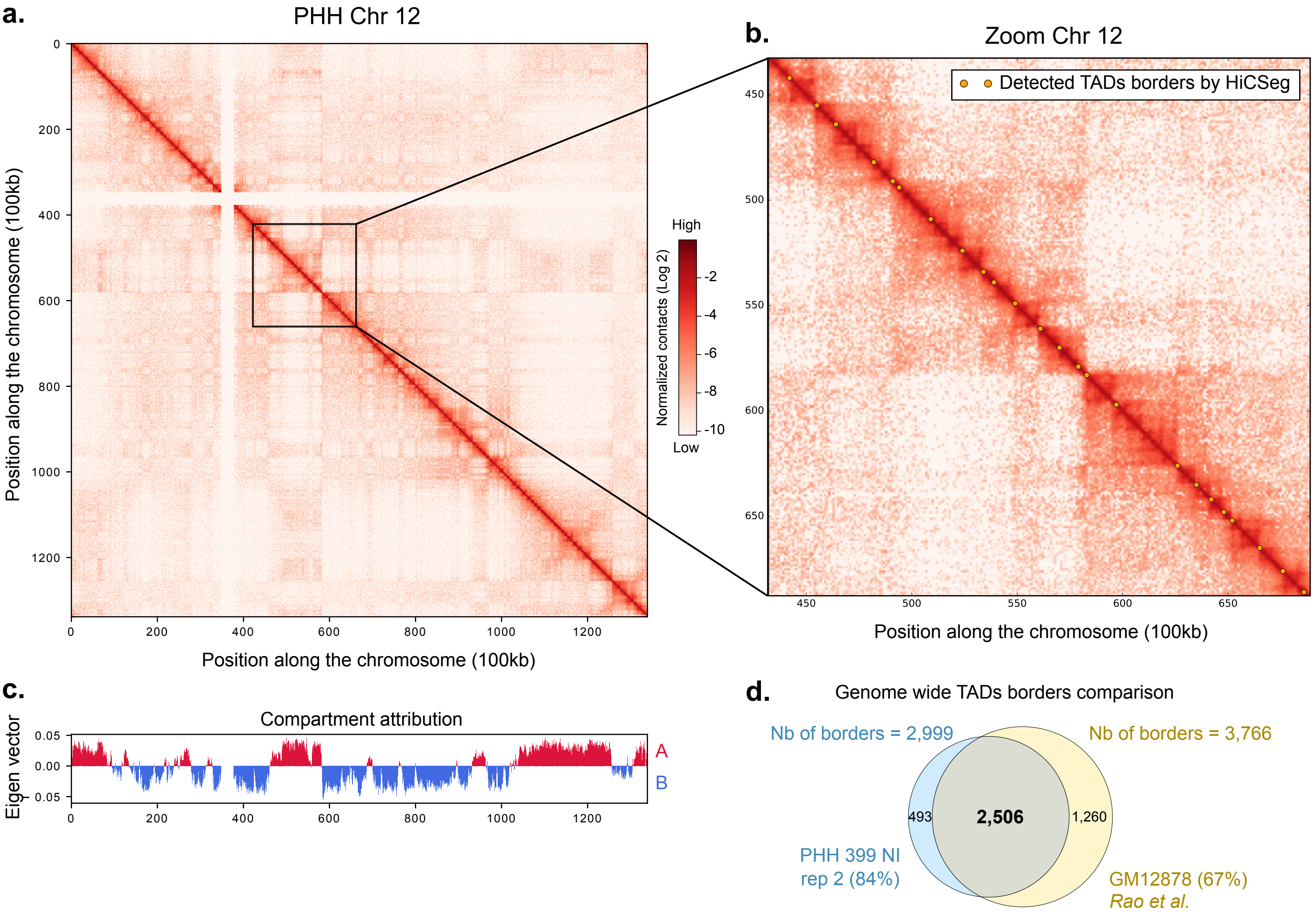

Supplementary Figure 2. (a) Hi-C contact maps of chromosome 12 from non-infected PHH at 100 kb resolution. (b) Example of TADs borders detection by Hi-CSeg, zoom of contact map of chromosome 12 at 100kb resolution. Each border is represented on the main diagonal with an orange dot (c) First principal component of chromosome 12 (100 kb resolution) representing the active A-type (red) and repressed B-type compartmentalization (blue). (d) Comparison of TADs borders at 100 kb between PHH non-infected and GM12878 cell line from Rao et al. [6] represented by a Venndiagram.

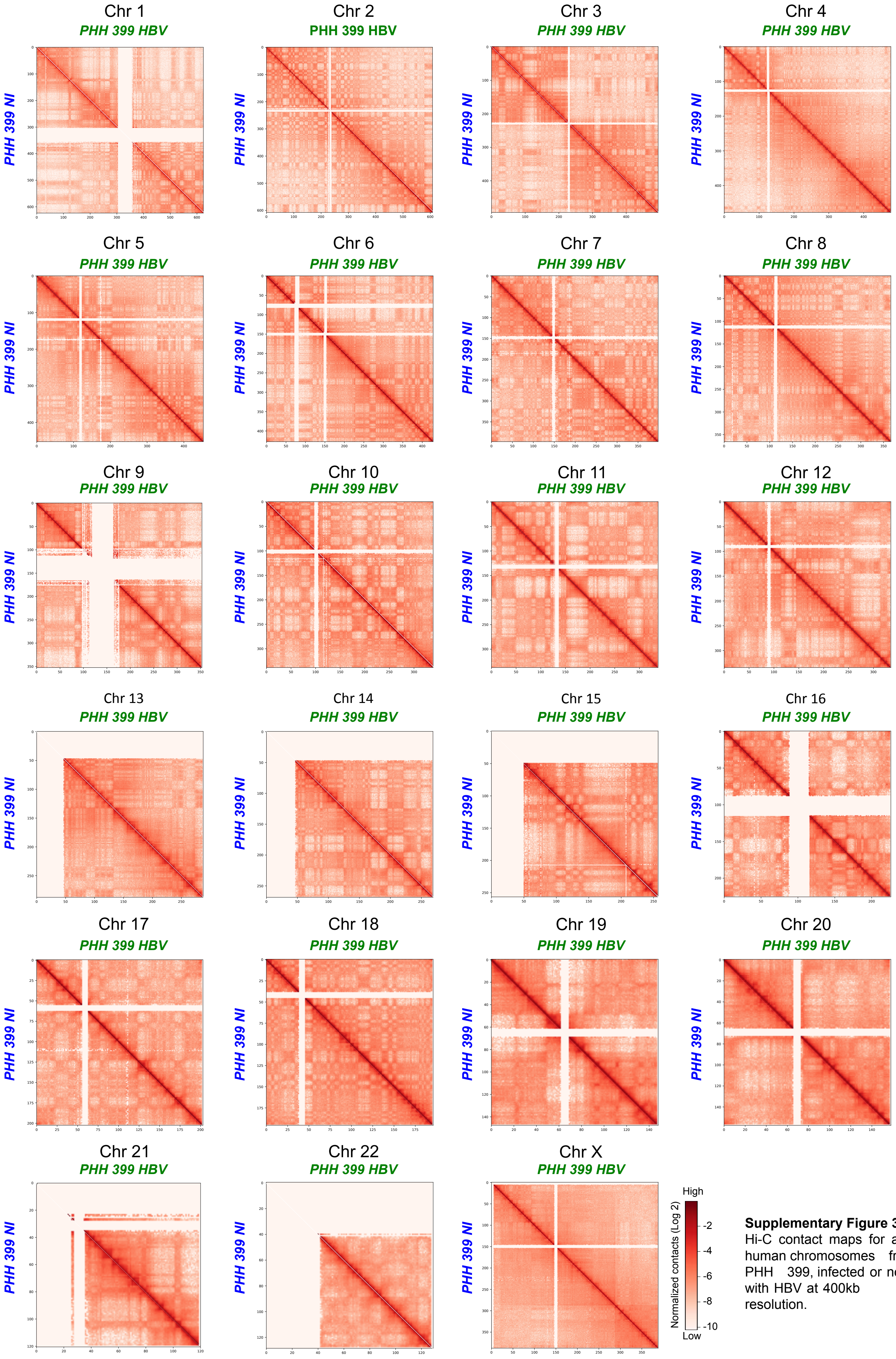

Supplementary Figure 3. Hi-C contact maps for all human chromosomes from PHH 399, infected or not with HBV at 400kb resolution.

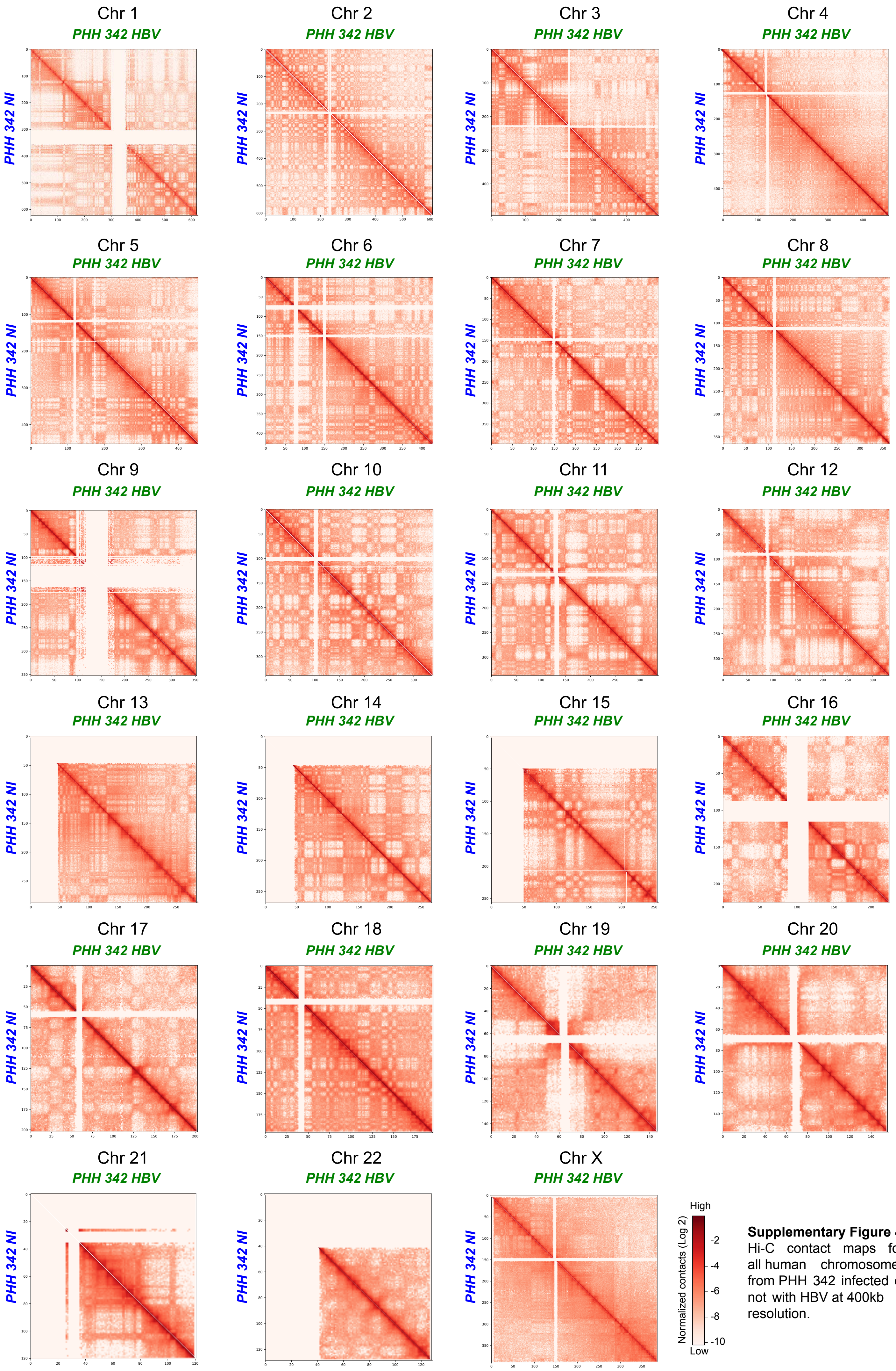

Supplementary Figure 4. Hi-C contact maps for all human chromosomes from PHH 342 infected or not with HBV at 400kb resolution.

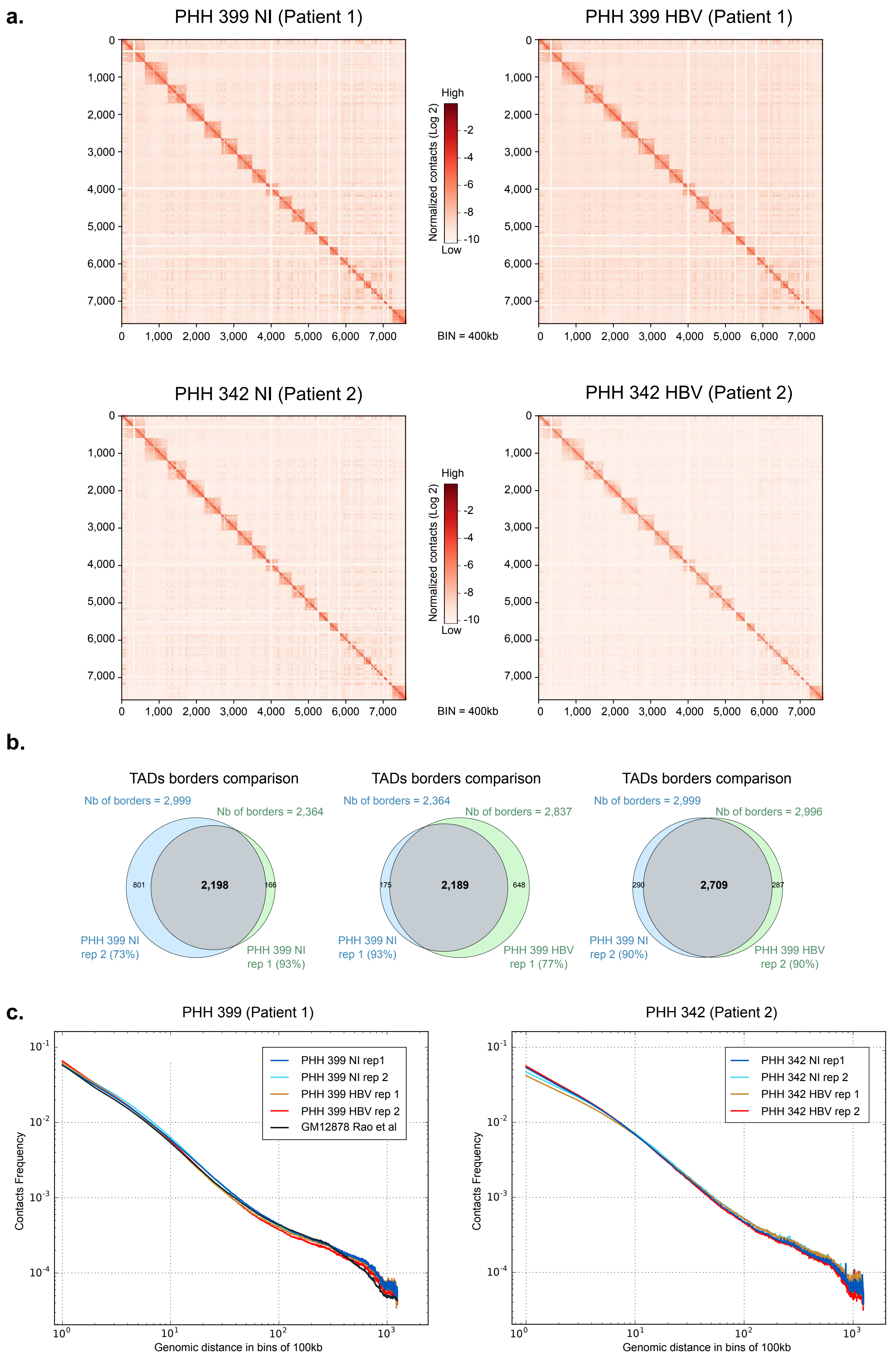

Supplementary Figure 5. (a) Genome-wide Hi-C contact map from PHH 342 and PHH 399 infected or not with HBV at 400kb resolution. (b) Comparison of TADs borders at 100kb between PHH grown using different culture conditions represented by Venndiagram. (c) Frequency of contacts in function of genomic distance at 100kb is identical in both non infected and HBV infected PHH 399 and 342. Also, Frequency of contacts in function of genomic distance at 100kb resolution is identical between PHH and GM12878 cell.

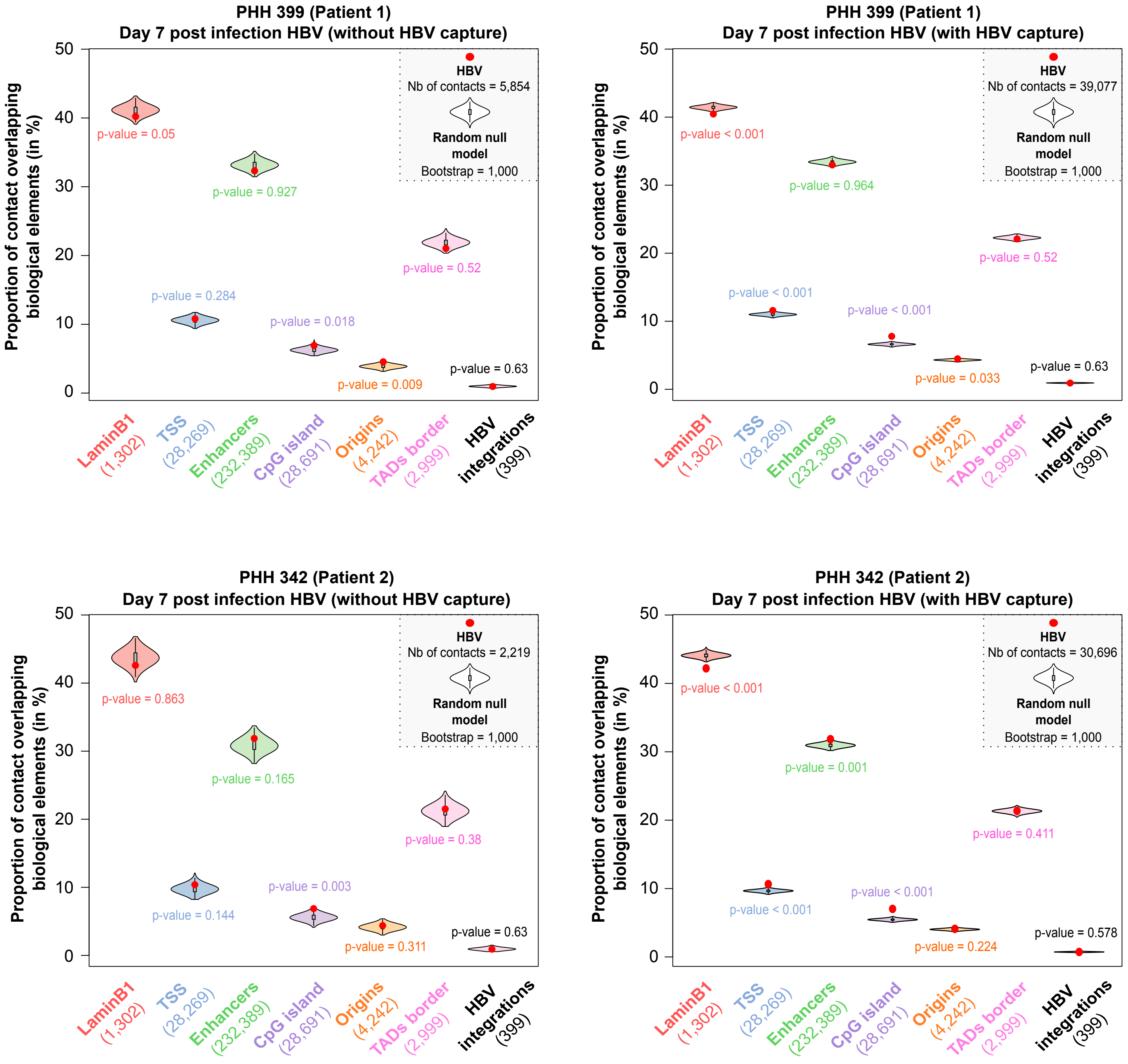

Supplementary Figure 6. (a) Proportion of HBV contacts, identified in PHH 399 infected by HBV, with a given biological element (as indicated on the graph) compared to the corresponding null model that shows the distribution using the Hi-C coverage and represented by violin plot. Density plot width = frequency, line = 95% confidence interval, box plot = interquartile range. Red dots represent the proportion of HBV contacts with the indicated biological element (computed in windows ± 3.5 kb around the start of the read). P-values were determined according to the percentages detected in the bootstrap strategy (see Methods). Left graph, the analysis was done using HBV contacts identified directly from HI-C libraries sequencing. Right graph, analysis was performed on HBV contacts enriched through HBV-capture. (b) similar to panel (a) for PHH 342.

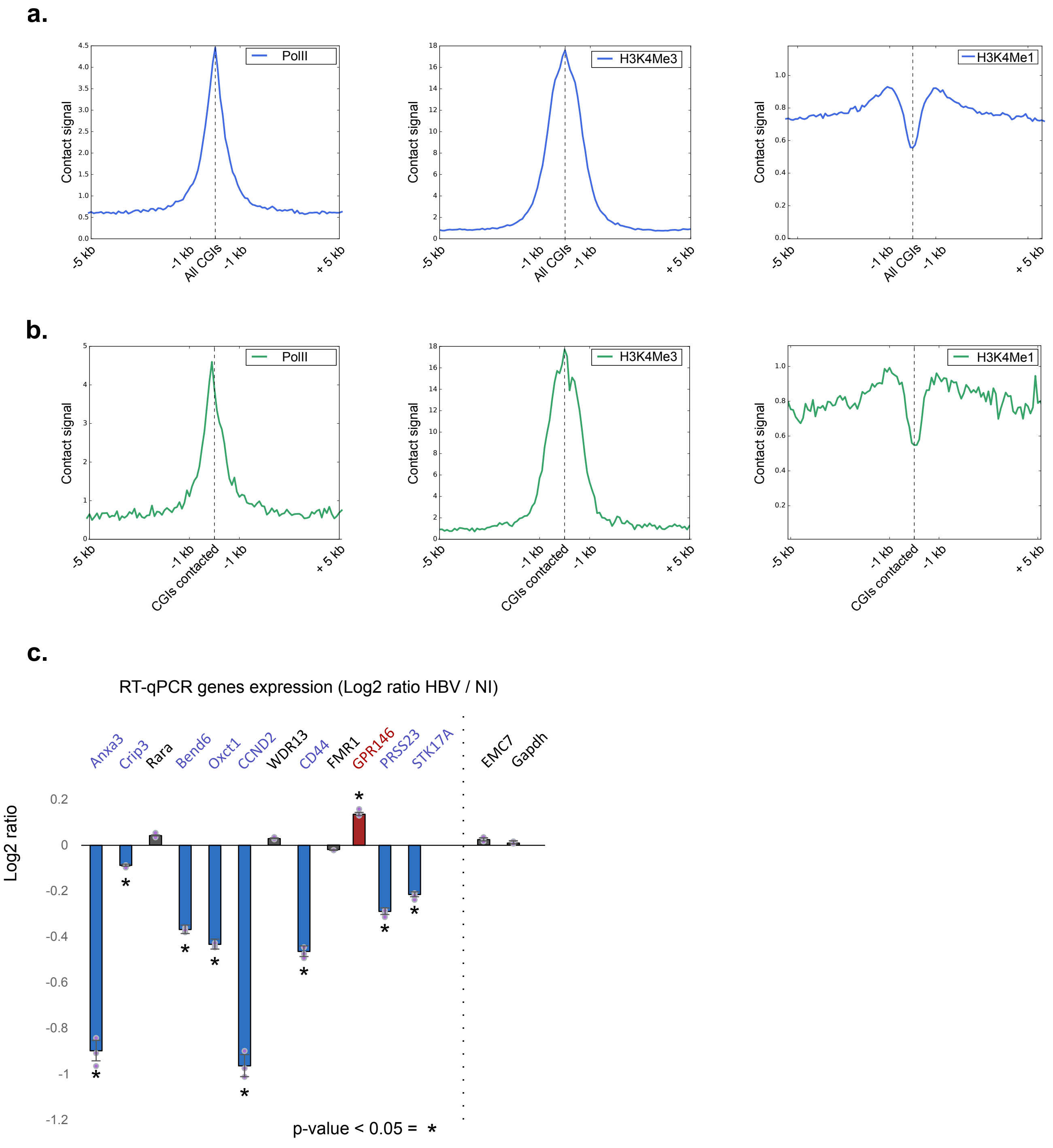

Supplementary Figure 7. (a) Enrichment plot for Pol II, H3K4me3 and H3k4me1 using ChIP-seq signal generated from Encode project from HepG2 cells (Encode Project:GSE29611) [17]. (b) Enrichment plot for Pol II, H3K4me3 and H3k4me1 by ChIP-seq signal at CGIs contacted by HBV (Encode Project:GSE29611) [17]. (c) Analysis of the expression of genes proximal to CGi contacted by HBV. PHH were infected at a MOI of 500 genomes equivalent/cells and treated during the last 3 days with lamivudine. 7 days after infection total RNA was prepared and the expression of the indicated cellular genes was quantified using RT-qPCR. Error bars (n = 3) represent SEM. *, p-values < 0.05 by Wilcoxon-Mann-Whitney test.

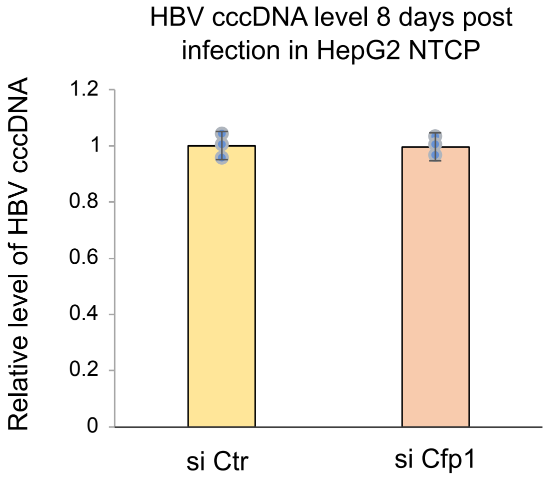

Supplementary Figure 8. HepG2 NTCP cells were infected with HBVwt at MOI of 100 vp/cell and transfected 24h later with siRNA control (si ctr) or directed against Cfp1. The cccDNA level in each condition was analyzed by qPCR. The cccDNA level in si Ctr HepaRG cells infected with HBV wt was set at 1. Error bars represent SEM of three independent experiments (n = 3).

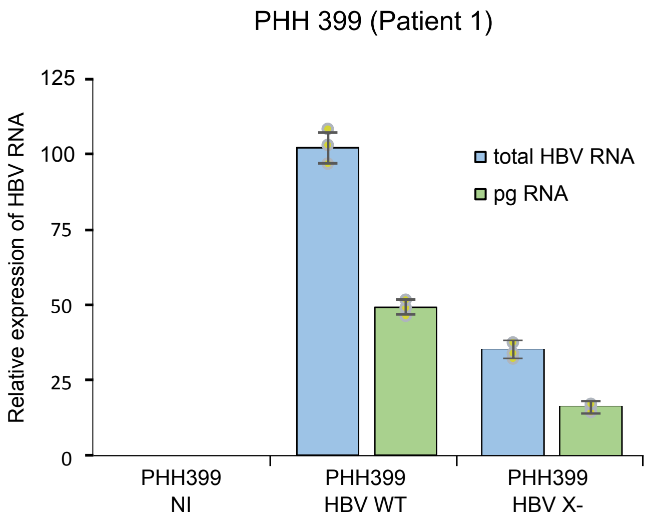

Supplementary Figure 9. PHH (donor 399) were infected at a MOI of 500 genomes vp/cells with HBV wild type (WT) or HBV X- and treated with lamivudine the last 3 days of the experiment. 7 days after infection cells were harvested and analyzed for HBV replication. HBV WT and X- transcription was assessed by the quantification of total HBV RNA and pgRNA by RT-qPCR. Error bars represent SEM of three independent experiments (n = 3).

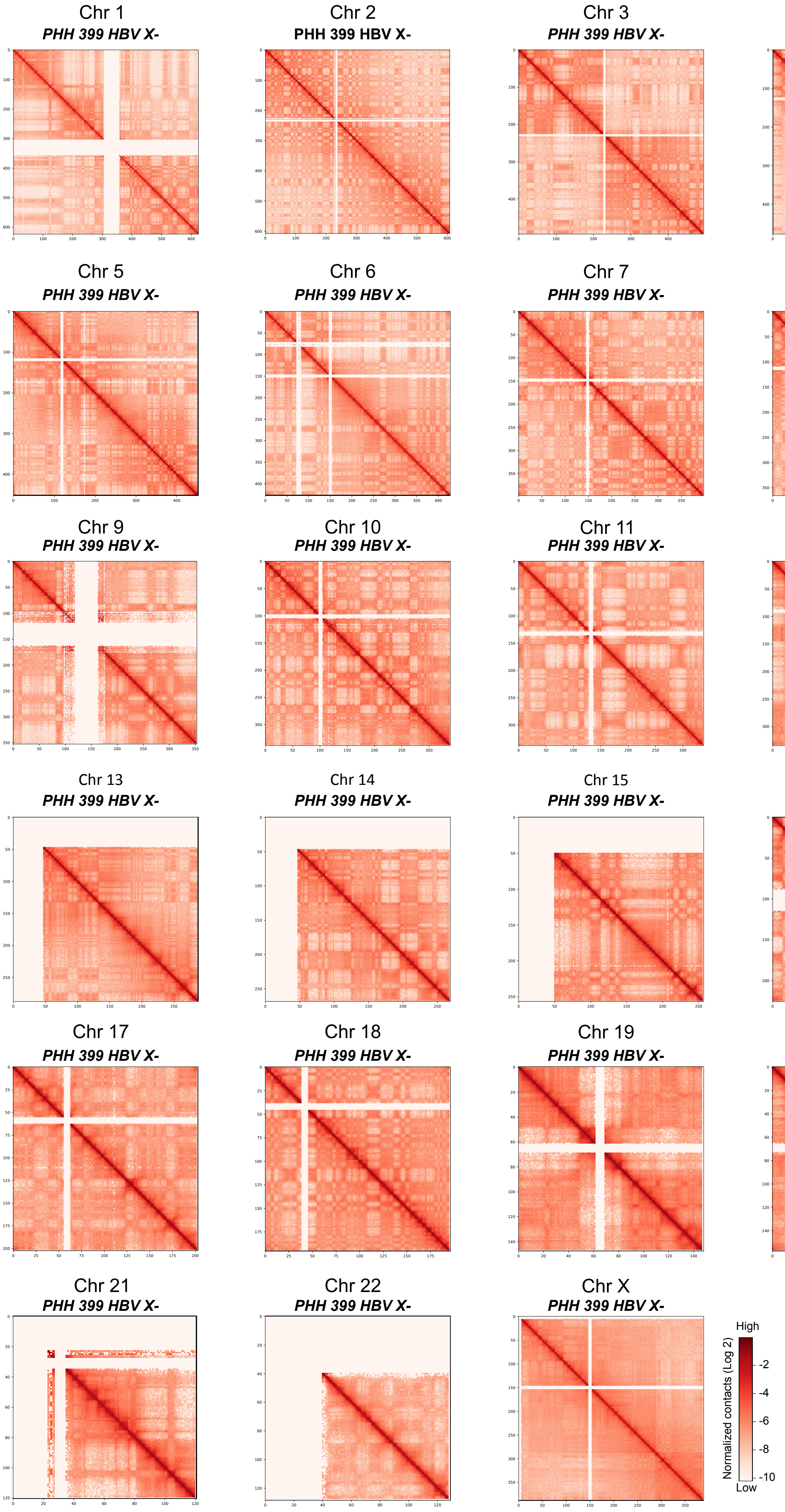

Supplementary Figure 10. Hi-C contact maps for all human chromosomes from PHH 399, infected with HBV X- at 400kb resolution.

a.

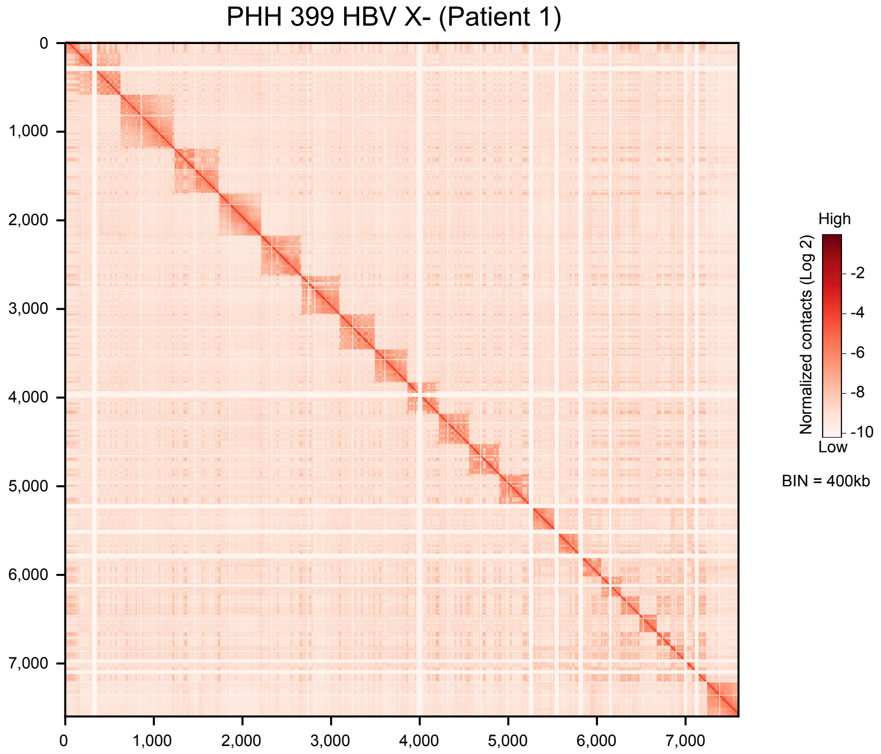

b.

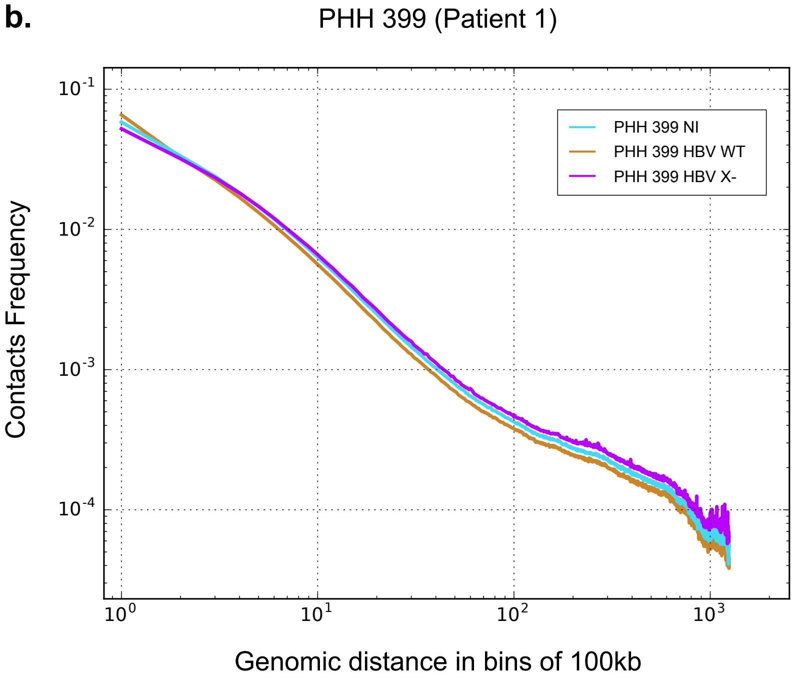

Supplementary Figure 11. (a) Genome-wide Hi-C contact map from PHH 399 infected with HBV X- at 400kb resolution. (b) Frequency of contacts in function of genomic distance is identical in non infected PHH and PHH 399 infected with HBVwt or HBV X- at 100kb resolution.

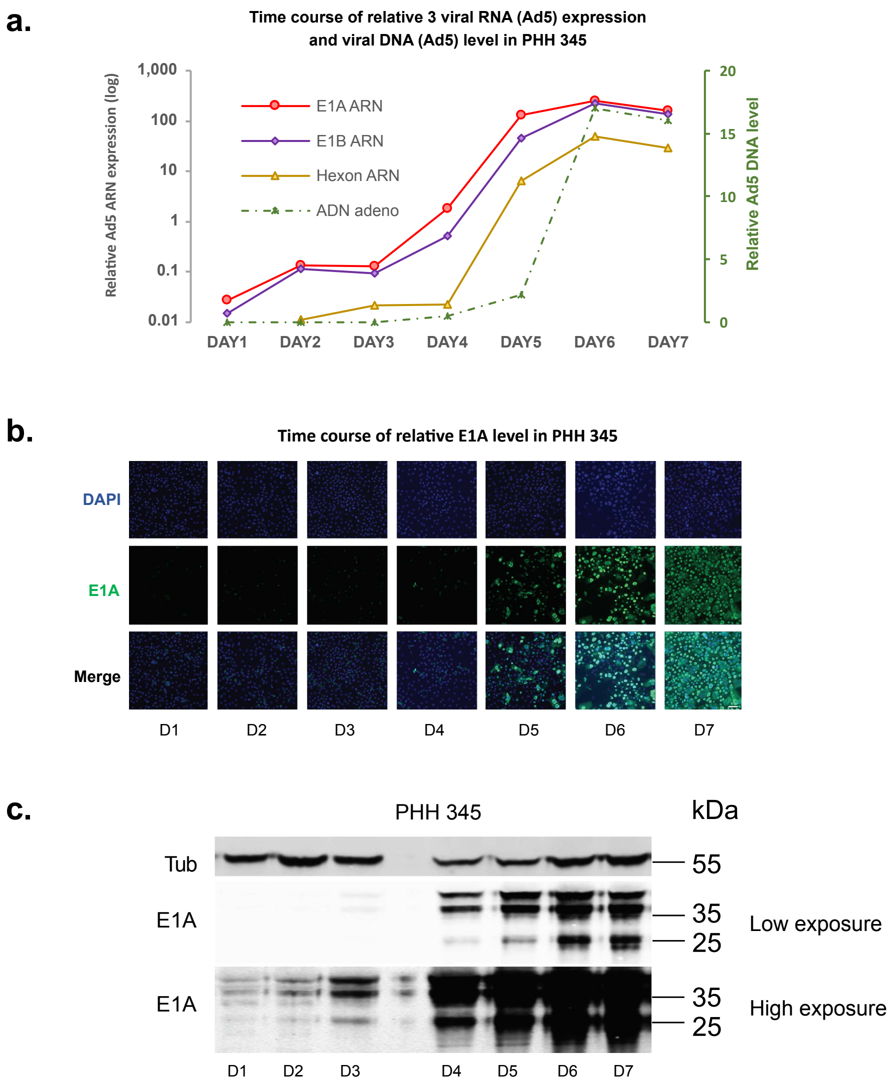

Supplementary Figure 12. PHH (345) was plated and harvested at the indicated time after plating. Either total RNA, cellular DNA or whole cell lysate were extracted or the cells were prepared for immunofluorescence. (a) Expression of two early transcripts (E1A and E1B) and one late transcript (Hexon also called protein II) were quantified by RT-qPCR. Ad5 replication was assessed by quantification of viral DNA using qPCR. (b, c) E1A expression was analyzed at the indicated time either by immunofluorescence, scale bar represents 50 µm (b) or by western blotting (c).

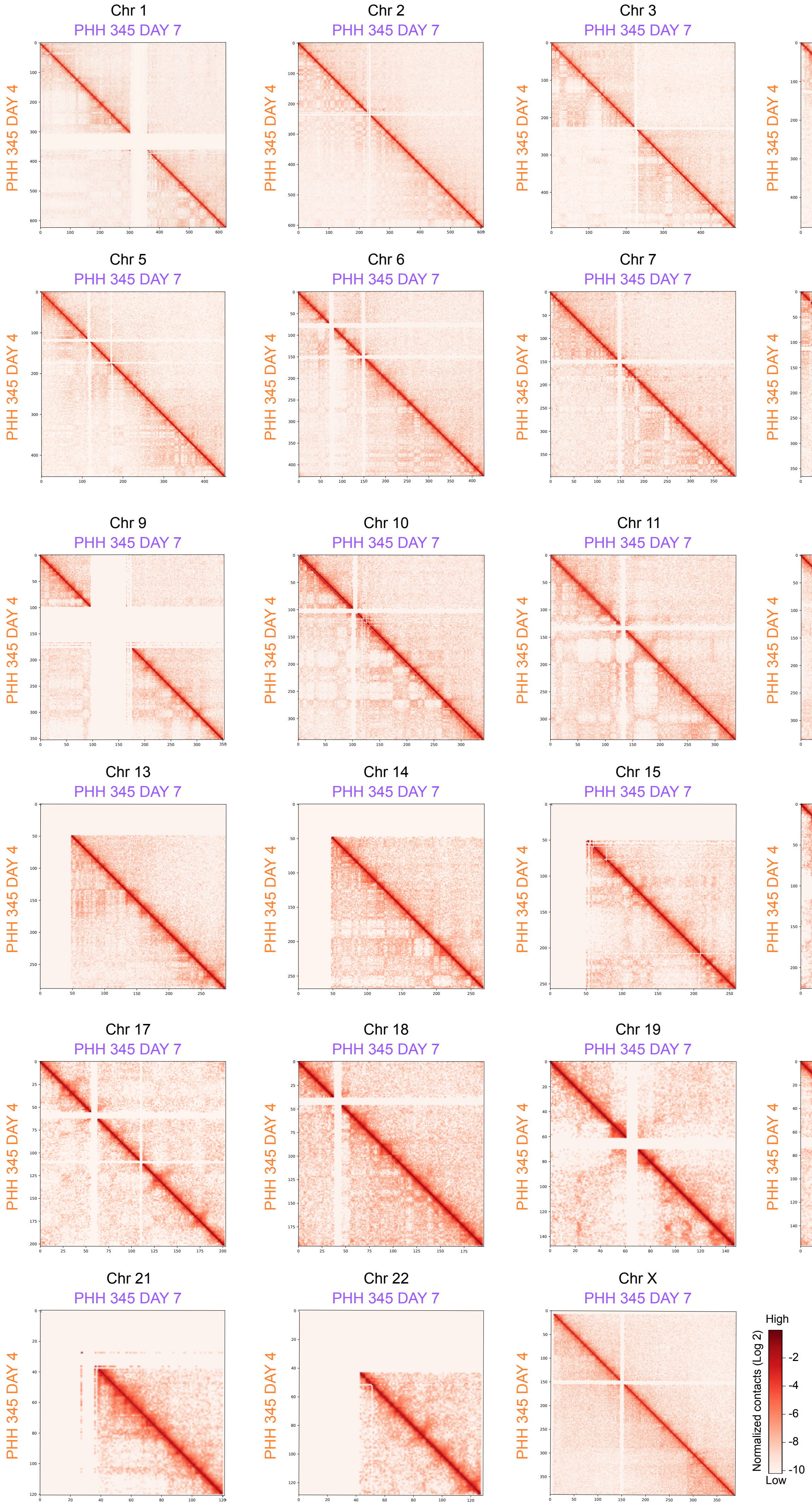

Supplementary Figure 13. Hi-C contact maps for all human chromosomes from PHH 345 day 4 vs day 7 at 400kb resolution.

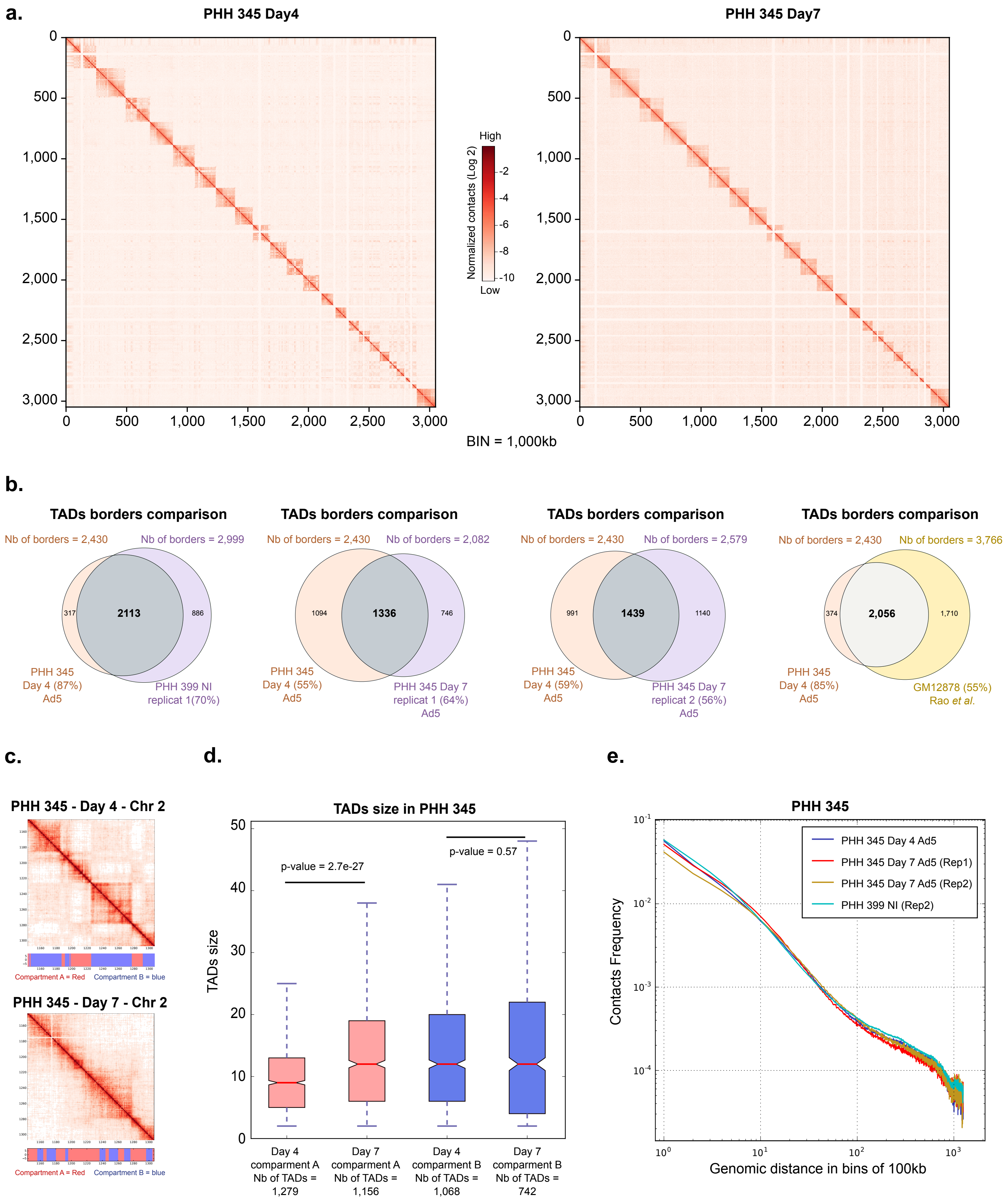

Supplementary Figure 14. (a) Hi-C contact maps for all human chromosomes from PHH 345 at day 4 or day 7 post-plating at 1,000kb resolution. (b) Comparison of TADs borders at 100kb between in PHH grown using different culture conditions and different cell lines represented by Venndiagram. (c) Zoom of Chr2 at 100kb to show TADs border comparison at day 4 and day 7 after plating (d) Quantification of TADs size for compartment A and B at day 4 and day 7 in all genome. Red line = median, box plot = interquartile range, line = 95% confidence intervale. p-values were determined by fisher test. (e) Frequency of contacts in function of genomic distance at 100kb is identical in PHH 399 NI, PHH 345 Day 4 and PHH 345 day 7.

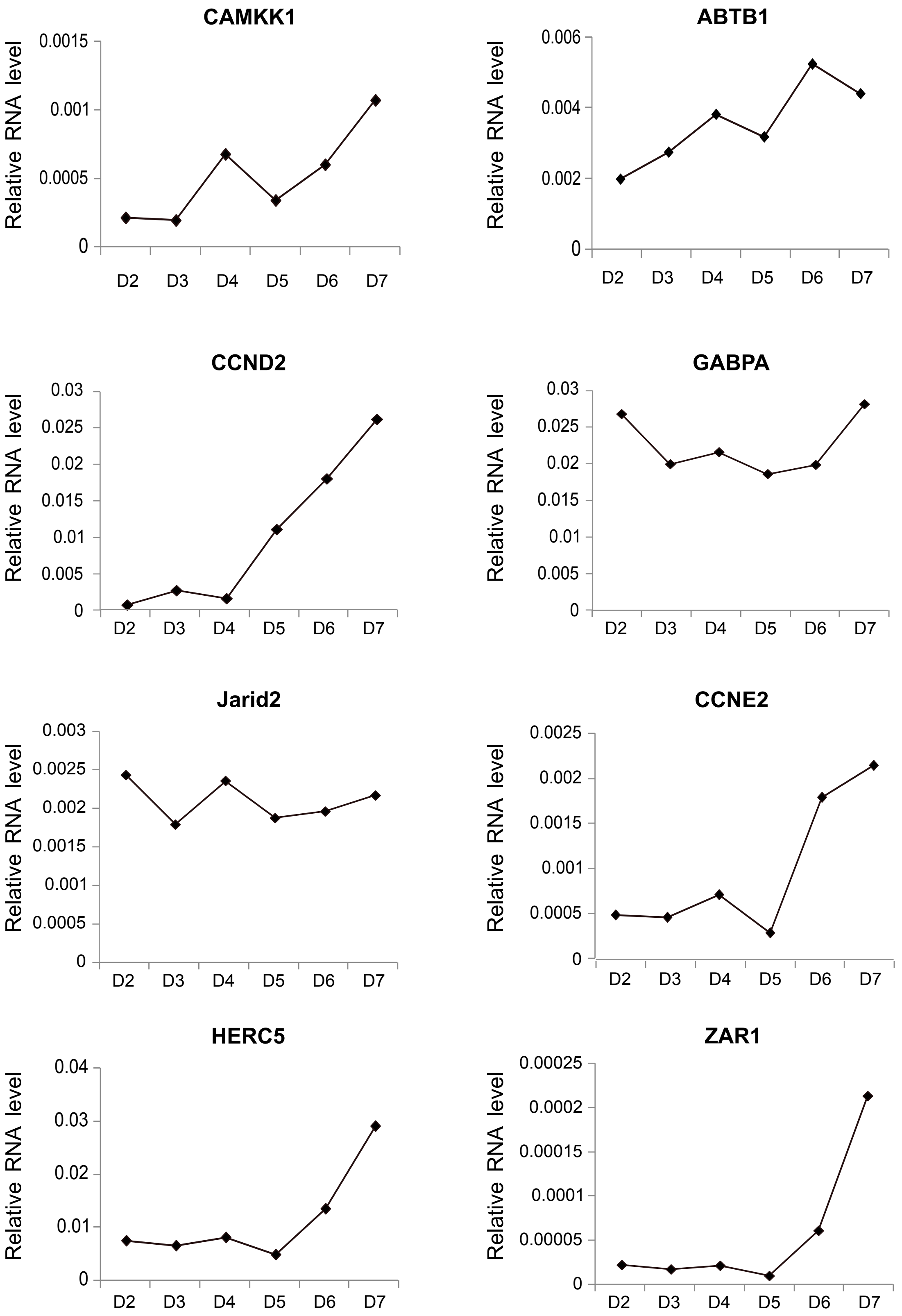

Supplementary Figure 15. PHH (345) were plated and total RNA was extracted at the indicated time post plating. The expression of the indicated cellular genes was quantified using RT-qPCR (n = 1).

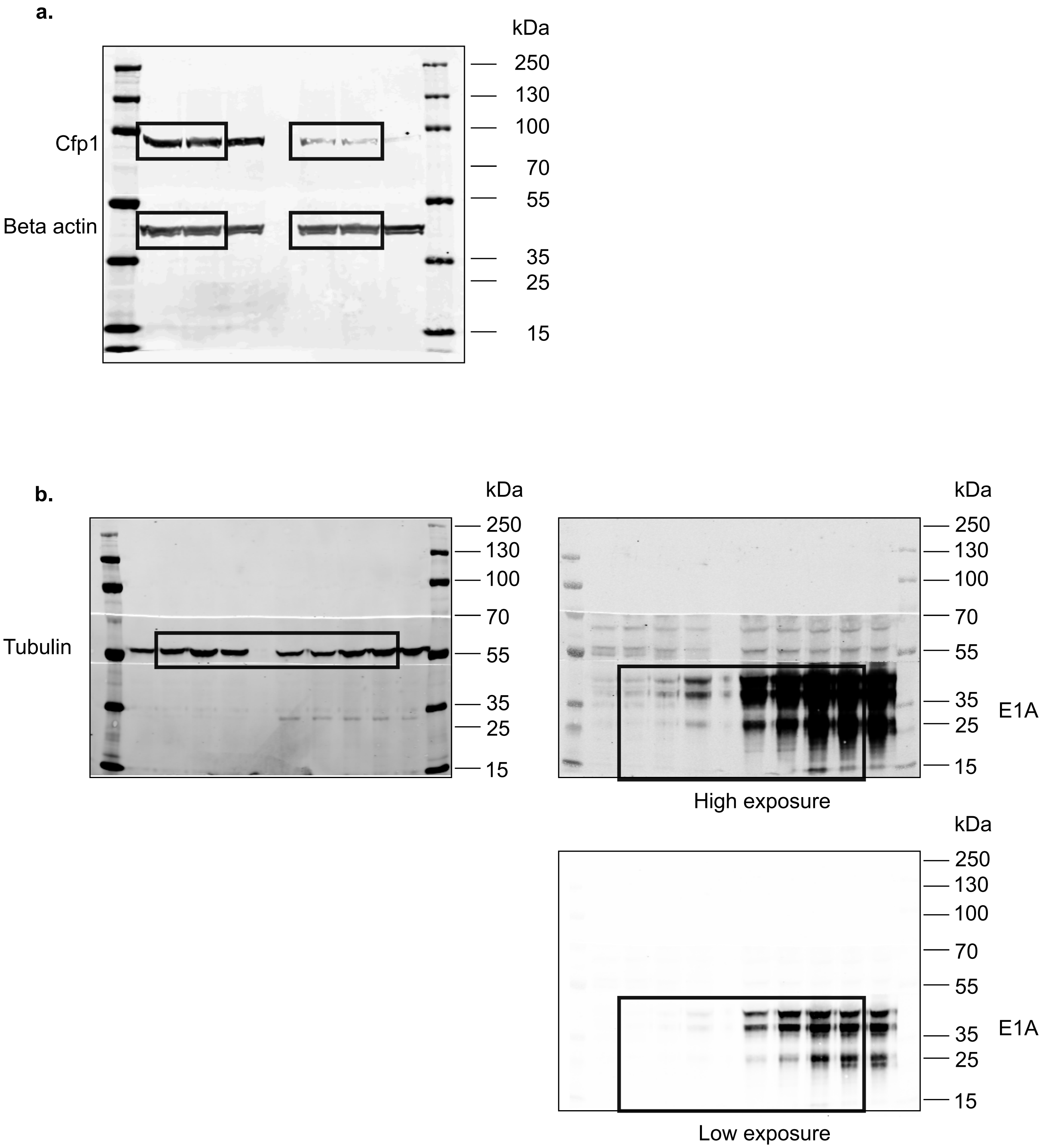

Supplementary Figure 16. Uncropped data for (a) Figure 5a and (b) Supplementary Figure 12c

Supplementary Table1. Hi-C table quality reads

	PHH399 NI	PHH399 NI	PHH399 HBV WT	PHH399 HBV WT	PHH342 NI	PHH342 NI	PHH342 HBV WT	PHH342 HBV WT	PHH399 HBV X-	PHH345 Day 4 p.p.	PHH345 Day 7 p.p.	PHH345 Day 7 p.p.
**Hi-C Sequence adapter**	BC162 Replicate 1	BC108 Replicate 2	BC164 Replicate 1	BC176 Replicate 2	BC180 Replicate 1	BC184 Replicate 2	BC182 Replicate 1	BC186 Replicate 2	BC78 Replicate 1	BC172 Replicate 1	BC176 Replicate 1	BC108 Replicate 2
**Genome reference**	hg19 + HBVayw	hg19 + HBVayw	hg19 + HBVayw	hg19 + HBVayw	hg19 + HBVayw	hg19 + HBVayw	hg19 + HBVayw	hg19 + HBVayw	hg19 + HBVayw	hg19 + Ad5	hg19 + Ad5	hg19 + Ad5
**raw read-pairs**	243,670,559	229,336,025	259,584,178	296,877,780	60,864,521	132,248,295	60,502,064	90,520,562	344,005,087	33,402,959	67,762,448	18,132,934
**Number of mapped read-pairs (MQ > 30)**	124,358,243	113,133,375	105,906,684	121,184,691	24,718,593	54,784,331	23,659,914	37,984,096	155,232,060	17,919,933	42,651,764	12,448,285
**Valid read-pairs**	97,101,646	91,699,108	68,259,589	92,676,866	21,885,060	48,725,102	18,793,708	27,950,685	115,731,917	15,949,771	24,835,347	9,029,013
**number of paired reads (after removal of PCR duplicates)**	30,244,950	53,827,338	26,977,467	60,691,913	17,425,540	39,902,700	13,324,944	24,110,987	88,861,245	5,197,119	7,046,449	4,394,128
**Viral - host genome contacts**	0	0	3,188	2,666	0	0	1,050	1,169	13,786	3,862	1,352,760	600,055
**% of viral - host interaction**	0	0	0,03%	0,01%	0	0	0,02%	0,02%	0.015%	0,30%	38,00%	32,00%
**Estimated number of virus copies per cell**	0	0	~60	~20	0	0	~40	~25	~75	~30	~9,100	~6,300
**CHi-C Sequence adapter**			BC164 Replicate 1	BC176 Replicate 2			BC182 Replicate 1	BC186 Replicate 2	BC78 Replicate 1			
**Genome reference**			hg19 + HBVayw	hg19 + HBVayw			hg19 + HBVayw	hg19 + HBVayw	hg19 + HBVayw			
**raw read-pairs**			4,597,183	19,275,280			6,669,530	13,763,195	51,520,169			
**Number of mapped read-pairs (MQ > 30)**			1,961,836	7,774,658			2,799,215	6,168,891	26,020,018			
**Valid read-pairs**			1,163,847	5,470,585			2,272,572	4,687,402	19,059,286			
**number of paired reads (after removal of PCR duplicates)**			819,000	4,251,749			2,128,746	4,386,185	16,307,405			
**Viral - host genome interactions with HBV capture**			21,690	11,533			12,755	15,725	106,735			
**% of viral - host interaction**			2,60%	0,27%			0,60%	0,36%	0,65%			
**Virus-human Enrichment (before / after capture)**			~ 90	~ 20			~ 30	~ 20	~ 43			

* estimated based on the ratio between reads coverage of virus over reads coverage on human genome

Supplementary Table 2. RNAseq table quality reads

Sample name	Raw reads	Clean reads	clean bases	Error rate(%)	Q20(%)	Q30(%)	GC content(%)
PHH_NI_1	111,124,614	106,267,562	15.9G	0.02	97.12	92.80	48.08
PHH_NI_3	91,196,966	88,092,422	13.2G	0.02	96.83	91.66	47.25
PHH_NI_4	97,001,690	93,400,592	14G	0.02	96.77	91.48	47.28
PHH_WT_1	115,577,544	11,0470,610	16.6G	0.02	96.87	92.25	48.50
PHH_WT_2	81,228,928	77,588,328	11.6G	0.01	97.90	94.50	47.30
PHH_WT_6	79,775,228	76,371,250	11.5G	0.01	97.88	94.46	47.63

Supplementary table3. Primers

Names	Sequence	Type
HBV RNAall-F	TGAACCTTTACCCCGTTGCC	RT-qPCR
HBV RNAall-R	GTATGGATCGGCAGAGGAGC	RT-qPCR
Pg RNA-F	TGTCAACACTAATATGGGCCTAA	RT-qPCR
Pg RNA-R	AGGGGCATTTGGTGGTCTAT	RT-qPCR
Rhot2-F	CTGCGGACTATCTCTCCCCTC	RT-qPCR
Rhot2-R	AAAAGGCTTTGCAGCTCCAC	RT-qPCR
HBV cccDNA-F	GTGCACTTCGCTTCACCTCT	ChIP-qPCR
HBV cccDNA-R	AGCTTGGAGGCTTGAACAGT	ChIP-qPCR
CCNA2-F'	CCTGCTCAGTTTCCTTTGGT	ChIP-qPCR
CCNA2-R	AGACGCCCAGAGATGCAG	ChIP-qPCR
RC-F	CACTCTATGGAAGGCGGGTA	IP preS1-qPCR
RC-R	TGCTCCAGCTCCTACCTTGT	IP preS1-qPCR
ANXA3 F	GTTGGACACCGAGGAACAGT	RT-qPCR
ANXA3 R	CACTAGGGCCACCATGAGAT	RT-qPCR
Crip3 F	GCCAGCAACCTGTTTTCTTC	RT-qPCR
Crip3 R	AGGAGCCTACACCACCAATG	RT-qPCR
RARA F	GTGTCACCGGGACAAGAACT	RT-qPCR
RARA R	CGTCAGCGTGTAGCTCTCAG	RT-qPCR
Bend 6 F	GCAGAAGATCGTGCAGACAG	RT-qPCR
Bend 6 R	GGCTCTTCATCCTCACTGGA	RT-qPCR
Oxct1 F	TCCTGGCCAGCAATTTTATC	RT-qPCR
Oxct1 R	CATCGCTCCTAGCATTGTCA	RT-qPCR
CCND2 F	TGGGGAAGTTGAAGTGGAAC	RT-qPCR
CCND2 R	ATCATCGACGGTGGGTACAT	RT-qPCR
WDR13 F	ACCGTGGCAGTGTCTTCTCT	RT-qPCR
WDR13 R	CTTGTTGAGGCAAGCATTGA	RT-qPCR
CD44 F	AAGGTGGAGCAAACACAACC	RT-qPCR
CD44 R	GCTTTTTCTTCTGCCCACA	RT-qPCR
FMR1 F	CACCTCAAAGCGAGCACATA	RT-qPCR
FMR1 R	CAATAGCAGTGACCCCAGGT	RT-qPCR
GPR146 F	CTTTGTCAACATGGCAGTGG	RT-qPCR
GPR146 R	AGTGCACGCTCGATGTAGTG	RT-qPCR
PRSS23 F	ACTTACGAAGAGGCCAAGCA	RT-qPCR
PRSS23 R	GTCCTTCCCAAAAATGCTGA	RT-qPCR
STK17A F	TGAATCTCCATTGGGTGACA	RT-qPCR
STK17A R	CCACATATCTGTTGCCATGC	RT-qPCR
EMC7 F	TGCCCTATCCTCTCCAAATG	RT-qPCR
EMC7 R	ATTTCCCGTCTCATGTCAGG	RT-qPCR
Gapdh F	AGCCACATCGCTCAGACAC	RT-qPCR
Gapdh R	GCCCAATACGACCAAATCC	RT-qPCR
E1A F	GTGCCCCATTAAACCAGTTG	RT-qPCR
E1A R	GGCGTTTACAGCTCAAGTCC	RT-qPCR
E1B F	GAGGGTAACTCCAGGGTGCG	RT-qPCR
E1B R	TTTCACTAGCATGAAGCAACCACA	RT-qPCR
Hexon F	GAACGGTGTGCGCAGGTA	RT-qPCR & qPCR
Hexon R	CGCTGGACATGACTTTTGAG	RT-qPCR & qPCR
CAMKK1 F	GACATCAAGCCATCCAACCT	RT-qPCR
CAMKK1 R	GGGCACTTCCCATAGACAAA	RT-qPCR
ABTB1 F	GGCTCTACGCGATTACAAGC	RT-qPCR
ABTB1 R	ATCAGTGGGTGCCTGAGAAC	RT-qPCR
CCND2 F	TGGGGAAGTTGAAGTGGAAC	RT-qPCR
CCND2 R	ATCATCGACGGTGGGTACAT	RT-qPCR
GABPA F	AAGTGACAAGATGGGCTGCT	RT-qPCR
GABPA R	CCGAAATGTTGAGTGTGGTG	RT-qPCR
Jarid2 F	TCCAAAGTGTGCTGTGGGTA	RT-qPCR
Jarid2 R	GCTGCCGTAGCTCTGTATCC	RT-qPCR
CCNE2 F	CAGGTTTGGAGTGGGACAGT	RT-qPCR
CCNE2 R	GGAAGAAATCATGCACAGCA	RT-qPCR
HERC5 F	GATTGCTGGAGGGAATCAAA	RT-qPCR
HERC5 R	TTGGATTTCCCTTTTTGTGC	RT-qPCR
ZAR1 F	CACTGCAAGGACTGCAACAT	RT-qPCR
ZAR1 R	GGCAGGAACATCTCGTTTGT	RT-qPCR
