# Supplementary material for: Tridimensional infiltration of DNA viruses into the host genome shows preferential contact with active chromatin
Source: Nat Commun. 2018 Oct 15;9:4268. doi: 10.1038/s41467-018-06739-4 (PMC6189100; doi:10.1038/s41467-018-06739-4)
Supplement: Supplementary file 3 — Description of Additional Supplementary Files [file 41467_2018_6739_MOESM3_ESM.pdf]

Description of Additional Supplementary Files

File Name: Supplementary Data 1
Description: (a) Functional annotation enrichment analysis of the genes containing CpG islands contacted by HBV genome. (b) List of the genes containing CpG islands contacted by HBV genome and used for the analysis

File Name: Supplementary Data 2
Description: Up regulated mRNA identified in RNAseq

File Name: Supplementary Data 3
Description: Down-regulated mRNA identified in RNAseq

File Name: Supplementary Data 4
Description: Up-regulated LNCRNA identified in RNAseq

File Name: Supplementary Data 5
Description: Down-regulated LNCRNA identified in RNAseq
